# Annual Meeting of Governors and General Council

**Published:** 1916-06-03

**Authors:** 


### KING EDWARD'S HOSPITAL FUND FOR LONDON.

### Annual Meeting of Governors and General Council.

The annual meeting of the Governors and General Council of King Edward's Hospital Fund for London to receive the accounts and the report of the General Council for the year 1915 was held at St. James's Palace on May 24, the Speaker of the House of Commons being in the chair.

Those present were: The Viscount Iveagh, the Chief Rabbi (the Rev. J. H. Hertz, D.D.), the Earl of Bessborough, the Viscount Mersey, Lord Revelstoke, Lord Farquhar, Lord Stamfordham, the Hon. Arthur Stanley, M.P., the President of the Royal College of Physicians (Dr. Frederick Taylor), the President of the Royal College of Surgeons (Sir W. Watson Cheyne, Bart.), the Rt. Hon. C. Stuart-Wortley, K.C., M.P., the Rt. Hon. Sir Savile Crossley, Bart. (Hon. Secretary), the Rt. Hon. Sir T. Vezey Strong, Sir William S. Church, Bart., Sir Henry Burdett, Sir Owen Philipps, Sir William H. Bennett, Sir William J. Collins, Sir Horace Marshall, Sir John Craggs, Sir John Tweedy, Mr. Frederick M. Fry (Hon. Secretary), Mr. John G. Griffiths (Hon. Secretary).

### The Year's Finance.

Lord Revelstoke (the Honorary Treasurer), in presenting the account of receipts and expenditure and the balance-sheet for the year ending December 31, 1915, said:

Mr. Speaker, my Lords and Gentlemen,—The account of income and expenditure is sufficiently analysed in the draft report which is before the Council to-day. I will only add a few remarks on matters with which the Finance Committee is specially concerned. The income from investments is larger than ever before. This is primarily due to the receipt of a full year's income from the first instalments of the Wernher legacy. I might perhaps mention that out of the whole of the revenue from the investments under the care of the Finance Committee, a revenue amounting to £97,000, the total loss to the income of the year through failure to pay interest or dividends since the war began amounts to less than £800, and this diminution of income is due to the non-receipt of interest or dividends on certain securities which were acquired by the fund as gifts or legacies from various donors, and for the purchase of which the Finance Committee were therefore not responsible. The Council will notice that, after this income from investments is taken into account, a sum of about £50,000 has still to be raised in order to maintain the distribution at last year's figure, without allowing for any increase in the needs of the hospitals.

The Council may like to hear that the Fund held on December 31 a large amount of War Stock. Of this the greater part represents investments, while the balance was derived from the conversion of Consols received from various executors in the past. I am glad to be able to state that the realised depreciation of £6,046 on trustee stocks, due to this conversion of Consols, was very nearly balanced by a realised appreciation on non-trust investments, leaving a net realised depreciation of only £262. At the present moment the Fund also holds large amounts of Treasury Bills and Exchequer Bonds.

### A Munificent Bequest.

Since the beginning of this year we have been notified by Sir Walter Trower of the very munificent bequest of the residue of the estate of the late Dowager Lady Wilton. The total residue is estimated to be about £150,000, and we have already received £100,000 on account. In deciding how to apply this large sum the Council will have to consider not only the general policy of the Fund in relation to legacies, but also the special conditions arising or likely to arise out of the war. The question will not come up until the autumn, and in the meantime the Finance Committee propose to deal temporarily with the cash to the best of their ability, without prejudice to any future decision which may be taken by the Council.

### The Council's Report.

Sir Savile Crossley (Hon. Secretary) presented the draft report of the Council for the year 1915. Its adoption was formally moved by the Speaker of the House of Commons, seconded by the Earl of Bessborough, and carried unanimously. The report is as follows:—

The total receipts for the year 1915 were £226,779 19s. 5d., of which £12,949 17s. 9d. were contributions to capital and £213,830 1s. 8d. receipts on general account. The General Fund receipts were made up as follows: Annual subscriptions, £25,724 16s. 11d.; donations, £5,360 16s. 3d.; contribution from League of Mercy, £14,000; special donation from Sir Ernest Cassel (4½ per cent. War Stock), £28,000; from the Lewis Estate, £34,121 8s. 1d.; other legacies, £4,412 10s. 7d.; dividends and interest from investments, £97,218 1s. 9d.; dividends and interest collected in 1915 but relating to 1914, £4,792 8s. 1d.; from the trustees of the Bawden Fund, £200; making a total of £213,830 1s. 8d.

### Larger Grants. Sir Ernest Cassel's Gift.

The very generous donation of £28,000 from Sir Ernest Cassel, like his similar gift of £31,500 in 1911, formed part of a total of £50,000 given by him for distribution amongst hospitals in London and the provinces, this time in the form of 4½ per cent. War Stock, 1925-45. The proportion entrusted to this Fund was distributed, as before, amongst the institutions receiving grants from the Fund, in the form of uppplementary grants equal in each case to one-fifth of the original grant. The effect of Sir Ernest Cassel's generosity was thus not only to increase the grants, but to ensure that a holding of War Stock should appear on the balance-sheet of each institution. The amount originally appropriated for distribution was £140,000, being the same amount as in 1914. Of this, £133,500 was allocated to London hospitals and £6,500 to consumption sanatoria and convalescent homes taking London patients. These totals were increased by the supplementary grants in respect of Sir Ernest Cassel's gift to £168,000: £160,200 and £7,800 to the two classes of institution respectively.

Of the £133,500 allocated by the Fund from its ordinary resources to the London hospitals, £111,775 was granted in aid of general maintenance, £5,750 to the reduction of debts on maintenance account, and £15,975 towards schemes of buildings or improvements: the supplementary grants following the same apportionment. Although the Fund has continued to encourage the hospitals in the policy of postponing all schemes of capital expenditure that were not already in hand at the outbreak of war or exceptionally urgent, the claims on the Fund in this respect were slightly greater than in 1914. Nevertheless, the amount granted in maintenance, even before the addition of the supplementary grants from Sir Ernest Cassel's special donation, was larger than in 1913.

**KING EDWARD'S HOSPITAL FUND**—(*continued*).

The £6,500 distributed by the Convalescent Homes Committee (apart from the supplementary grants of £1,300 War Stock) was allocated as follows: £4,560 to convalescent homes and £1,940 to consumption sanatoria. The increased grants to sanatoria enabled fifty-two beds to be reserved for the use of patients in London hospitals, as against forty-four last year. This increase was rendered possible by the fact that several convalescent homes formerly eligible as taking London patients had either been closed owing to the war or had become, for the time being, country hospitals for wounded. The question whether the sphere of operations of the Fund could be extended to include such hospitals, or certain other institutions now outside its limits, was very carefully considered, and it was decided that in view of the existing and prospective needs of the hospitals now within the area, no extension was advisable at the present time.

### Two Million Pounds Distributed.

The total sum distributed amongst hospitals, convalescent homes, and consumption sanatoria during the last ten years (after deducting conditional grants to the extent of £7,500 that have been allowed to lapse) is £1,484,750. Since the foundation of the Fund a total amount of £2,098,416 8s. 5d. has been distributed. The first million was passed in 1909, the thirteenth year of the Fund. It has taken six years for the grants to reach the second million. The average annual distribution for the whole period of nineteen years has been £110,442 19s. 4d.

During the year the amount spent on administration was £3,205 16s. 1d., or £1 8s. 3d. per cent. of the total amount received, as compared with £3,178 6s. 5d., or 12s. per cent., last year. The corresponding percentage for the whole period since the inauguration of the Fund has been £1 4s. 7½d., and the average yearly outlay in this respect has been £2,819 0s. 7d.

His Majesty the King, patron of the Fund, has been graciously pleased to continue his annual subscription of £1,000. Her Majesty Queen Mary, Her Majesty Queen Alexandra, His Royal Highness the Prince of Wales, and many other members of the Royal Family, have also again honoured the Fund with subscriptions.

The list of contributors includes annual subscriptions of £5,000 from Mr. Walter Morrison, £4,000 from the Drapers' Company, £1,000 from Lord Astor, and £1,000 from the Merchant Taylors' Company; besides an anonymous donation of £1,000. The Fund has also received annual subscriptions of £500 from the City Corporation, from Lord Iveagh, from Sir John R. Ellerman, Bart., from Mr. Robert Fleming, from Messrs. Morgan, Grenfell and Co., and from Messrs. N. M. Rothschild and Sons; and donations of the same amount from the Clothworkers' Company, and from "S.D.R.S.D."

The League of Mercy this year has again contributed £14,000, and the Council is greatly indebted to the members of the League for so successful an effort during a time of special difficulty. The total amount received from the League of Mercy since its foundation in 1899 now amounts to £230,000, apart from the sums awarded by the League to institutions outside the area of the Fund's distribution. The Council desire to express their appreciation of this very substantial benefit derived from the efforts of a numerous band of voluntary workers.

#### LEGACIES IN 1915.

Legacies received during the year include (in addition to a further amount of £34,121 8s. 1d. from the Lewis bequest) £6,174 12s. 9d., on account of the residue of the estate of the late Mr. Alfred John Heath, and an additional sum of £1,182 9s. 5d. given by the executors of the late Miss Emma Brandreth in the exercise of their discretionary powers. The Fund has also received the sum of £475, "a payment by the residuary legatee, Miss Letitia Overend, to carry out what she believed to be the wish and intention of her late uncle, Mr. T. B. G. Overend." The amount announced in the last annual report as received from the estate of the late Sir Julius Wernher was increased by further receipts of £6,300 during the year to £320,015 13s. 2d. The very generous legacy of one-twelfth of the residue of his estate, of which this represents the first instalments, was left in augmentation of the capital account of the Fund; and the interest has been of the greatest value in helping to maintain the distribution. Since the end of the year the Fund has received notification of the munificent bequest of the residue of the estate of the late Isabella Dowager Countess of Wilton. This legacy, which is estimated at £150,000, will very greatly assist the Fund to provide for the exceptional demands which it is feared may be made upon it in the near future. In order to maintain the distribution on the same scale as that of the last two years, an annual sum of nearly £50,000 has to be raised from subscriptions, donations, and legacies, and as there is every reason to believe that the full effect of the war, both upon income and upon expenditure, has still to be felt by the hospitals, the claims on the Fund are likely to increase rather than to diminish.

The total amount received from annual subscriptions and donations during 1915 (apart from the gift of Sir Ernest Cassel) showed, as compared with 1914, a decrease of £162 8s. 4d., while the receipts from legacies (other than the Lewis and Harman bequests) were £24,083 18s. 8d. less than in the previous year, and were £27,482 6s. 10d. less than the average of the five years 1910-1914.

### The Work of the Year.

The Executive Committee of the Fund have kept themselves informed on various matters arising out of the war which affected the London hospitals upon which the action of a central Fund such as this could be of assistance. Amongst other matters the committee have considered and corresponded with the authorities on the formulation of a scale of grants-in-aid in respect of hospital treatment of naval or military patients, which is now in general operation; and on the deficiency of resident medical officers resulting from the requirements of the Army and Navy Medical Services.

The inquiry into the subject of pensions for hospital officers was delayed at the beginning of the war. But the preparation of the report of the sub-committee was resumed later on, and was still in progress at the end of the year.

The statistical report on the expenditure of the London hospitals has been continued during the war in a modified form. All calculations that do not seem to be of sufficient value under the exceptional circumstances have been omitted, at some saving of cost and labour, while particulars relating to naval and military patients have been added.

Each eligible hospital and consumption sanatorium which applied for a grant was inspected and reported upon, as hitherto, by two of the Fund's visitors, one medical man and one layman in each case.

**KING EDWARD'S HOSPITAL FUND**—(*continued*).

At the annual meeting of the Governors and General Council, held at St. James's Palace on April 19, 1915, the following resolution was unanimously passed :

"That the Governors and General Council desire to place on record their profound regret at the death of Lord Rothschild, Hon. Treasurer of the Fund since its foundation. They recall with gratitude the great services rendered by him as Treasurer and as an active member of the Council during the whole period of its existence, as trustee and member of the Executive Committee down to the incorporation of the Fund by Act of Parliament in 1907, and subsequently as Chairman of the Finance Committee. They are deeply indebted to Lord Rothschild for the skill and care which he devoted to the ever-growing responsibilities of his office, and they deplore the loss of a colleague whose name, whose ability, and whose interest in the work of charity were a constant source of strength to the Council and to the Fund."

The Council also regret to record the death of Mr. William Latham, K.C., who was a member of the Council and had been Chairman of the Convalescent Homes Committee from 1898 to 1914. The Fund has also lost through death the services of Mr. Stanley Boyd, F.R.C.S., Dr. G. A. Heron, F.R.C.P., and Mr. Edmund Owen, F.R.C.S., who had given valuable help as visitors on behalf of the Fund for many years.

The President of the Local Government Board, in pursuance of his powers under the Act, has appointed Messrs. Deloitte, Plender, Griffiths and Co., chartered accountants, to audit the accounts of the Fund for the year. They have been the honorary auditors of the Fund since its foundation.

The Council are under great obligations to the Press for the valuable services they have rendered in the past, and continue to rely upon their kindly co-operation in obtaining for the Fund wider publicity and support.

The Bland-Sutton Institute of Pathology : Apportionment of Expenses Approved.

Sir William Church (Chairman of the Distribution Committee) presented a report of that committee on the expenses of the Bland-Sutton Institute of Pathology at the Middlesex Hospital. The report recommended that the scheme of apportionment between hospital and medical school now submitted by the hospital should be accepted as regards the year 1915 as satisfying the condition attached to the grants made by the Fund in December last—namely, that the expenditure should be allocated in the accounts for 1915 in accordance with a scheme of apportionment to be approved by the Fund.

Sir William Church, in moving the adoption of the report, said that, with regard to the lengthy and intricate matter which the Distribution Committee had had to consider, he would like publicly to state how harmoniously they had worked with the Middlesex Hospital. They had several meetings with the representatives of the hospital, and themselves paid a visit to the hospital, and were always treated by them with the greatest courtesy; and, during the whole of the business which they had to examine into, the negotiations were of the most friendly character.

An Emergency Grant for the National Sanatorium Association.

In the absence of Dr. Freshfield (Chairman of the Convalescent Homes Committee), Sir William H. Bennett moved the adoption of a report of that committee on the subject of a special application from the National Association's Sanatorium at Benenden.

The report, which was seconded by the President of the Royal College of Surgeons (Sir W. Watson Cheyne) and adopted, recommended that the committee be empowered under certain circumstances to promise, and if required to pay over, an emergency grant of a limited amount.

The Chief Rabbi (the Rev. Dr. Hertz) proposed a vote of thanks to the Speaker of the House of Commons for presiding.

Acknowledgments to the Finance Committee.

Viscount Mersey, in seconding the resolution, said that it must have struck them all that the management of the funds which Lord Revelstoke and the Finance Committee of the Fund had in their care had been most admirable. The times we had been passing through were times of great stress and strain, particularly in connection with money matters. He thought it very much to the credit of the gentlemen to whom the monetary interests of this great Fund were entrusted that they were able to show such an admirable result at the end of their year of work.

The Speaker's Forecast.

The Speaker, in acknowledging the vote of thanks, said that he agreed with Lord Mersey as to the satisfactory character of the balance-sheet and statement of accounts. He thought they must prepare their minds for the fact that, as the war continued, the margin of eleemosynary funds available for the general charities of the country must shrink to a considerable extent. This margin had been largely drawn upon, and, as time went on, it would be still more largely drawn upon, and no doubt this Fund would be amongst the sufferers. Fortunately, up to the present, they had got through their difficulties extraordinarily well; but they must not be disappointed if, at the meeting next autumn or this time next year, they found a considerable shortage. We all had to suffer in this respect, and it was impossible to expect that this Fund would be immune from the ills which charitable flesh is heir to.

